# Wnts acting through canonical and noncanonical signaling pathways exert opposite effects on hippocampal synapse formation

**DOI:** 10.1186/1749-8104-3-32

**Published:** 2008-11-05

**Authors:** Elizabeth K Davis, Yimin Zou, Anirvan Ghosh

**Affiliations:** 1 Neurobiology Section, Division of Biological Sciences, UCSD, 9500 Gilman Drive, La Jolla, CA 92093-0366, USA

## Abstract

**Background:**

Wnt proteins comprise a large class of signaling molecules that regulate a variety of developmental processes, including synapse formation. Previous studies have shown Wnts to be involved in both the induction and prevention of synapses in a number of different organisms. However, it is not clear whether the influence of Wnts on synapses is a result of Wnts' behavior in different organisms or differences in the activity of different Wnt ligands.

**Results:**

We used *in situ *hybridization to show that several Wnt ligands (Wnt3, Wnt5a, Wnt7a, and Wnt7b) and their receptors, Frizzled, are expressed in the developing hippocampus during the period of synapse formation in rodents. We used recombinant Wnt protein or Wnt conditioned media to explore the effects of Wnts on synapses in hippocampal cultures. We found that Wnt7a and Wnt7b activate canonical signaling, whereas Wnt5a activates a noncanonical pathway. The activation of the canonical pathway, either through pathway manipulations or through Wnt stimulation, increases presynaptic inputs. In contrast, exposure to Wnt5a, which activates a noncanonical signaling pathway, decreases the number of presynaptic terminals.

**Conclusion:**

Our observations suggest that the pro- and antisynaptogenic effects of Wnt proteins are associated with the activation of the canonical and noncanonical Wnt signaling pathways.

## Background

Wnts comprise a large family of 19 different secreted proteins that have been described in numerous developmental processes. Wnts can signal through several different types of receptors, but the most widely recognized Wnt receptors are Frizzled proteins (Fzd). Fzds comprise a family of 10 different G-coupled protein receptors [1]. Wnts are able to elicit a variety of responses in the target cell through Fzd activation, but the best studied is the canonical signaling pathway. The canonical pathway begins with activation of Fzd and a low-density lipoprotein receptor-related protein (LRP)5/6 coreceptor, resulting in the phosphorylation of Disheveled (Dvl). Dvl then disrupts a complex of proteins that consists of Axin, APC, and glycogen synthase kinase (GSK)3β. This complex normally degrades β-catenin through phosphorylation, so when Wnts bind, β-catenin levels are stabilized. B-catenin is then actively transported to the nucleus to regulate transcription [2]. Several other noncanonical Wnt pathways that signal through Fzd have been identified, including the planar cell polarity pathway and the Wnt/Calcium pathway [3]. However, new Wnt signaling pathways that act through Fzd or various other receptors are emerging [4].

Several observations suggest that Wnt proteins are involved in synapse formation. First, many Wnt signaling components are present at the synapse [5,6]. Second, many studies have implicated Wnts in synapse formation, both as pro- [7-9] and antisynaptogenic [10,11] molecules. In vertebrates, Wnts promote growth cone expansion and increase presynaptic puncta formation [7,8]. In *Drosophila*, Wnts act both pre- and postsynaptically to positively regulate presynaptic bouton number, presynaptic active zone assembly, and postsynaptic glutamate receptor distribution [9]. Conversely, in *Caenorhabditis elegans*, Wnts are antisynaptogenic molecules and prevent synapse formation near the tail of the animal [10]. It is not known whether these different effects of Wnt signaling reflect different roles of individual Wnt proteins or whether they reflect the activity of different Wnts in different organisms.

In this study we explore the function of Wnt proteins in synapse formation by examining the effects of several developmentally expressed Wnts on hippocampal cultures. We show here that several Wnts and Fzds are expressed in the developing hippocampus and that Wnts have differential synaptogenic activity according to the ability of Wnts to stabilize β-catenin.

## Results

### Wnts and Fzds are expressed in the hippocampus during synapse formation

**Figure 1:**
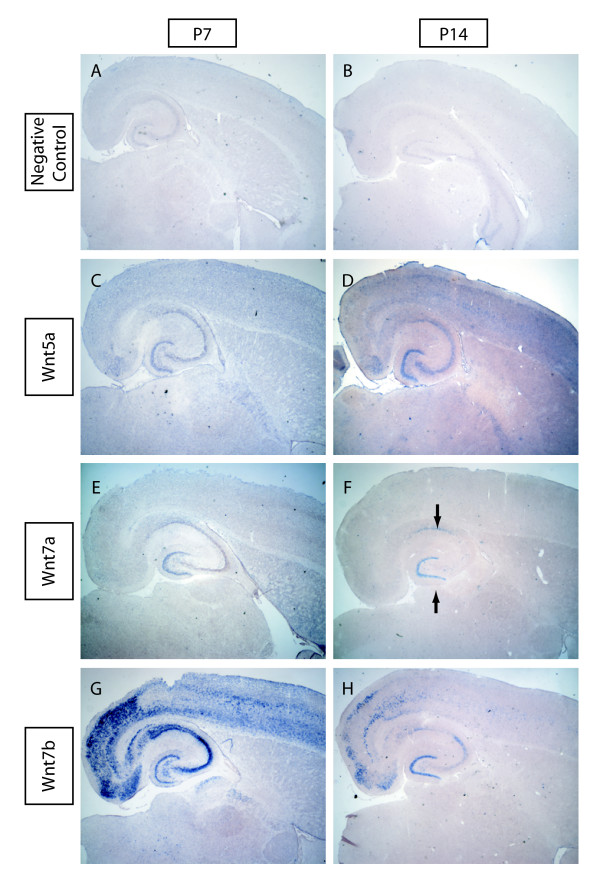
**Wnt expression in the developing hippocampus**. (A, B) Negative control *in situ *hybridizations at postnatal day 7 (P7) (A) and P14 (B). (C-H) Three Wnts are broadly expressed during synaptogenesis in the hippocampus. Wnt5a is expressed in all subregions at both P7 (C) and P14 (D). Wnt7a is expressed in the dentate gyrus at P7 (E) and in the dentate gyrus and CA1 regions at P14 (F; arrows). Wnt7b is expressed in all subregions at P7 (G) and P14 (H). Expression was determined using 20× magnification to differentiate signal from background.

To determine which Wnts might be involved in synapse formation, we performed an *in situ *hybridization screen to identify Wnts that are expressed in the hippocampus during synaptogenesis. In rodents, most synapses in the brain are formed within the first two weeks after birth [12,13]. We therefore chose to observe the expression of Wnts in horizontal mouse brain slices at postnatal day 7 (P7) and P14. Using DIG-labeled probes to all 19 Wnt genes, we found that only four, *Wnt5a*, *Wnt7a*, and *Wnt7b*, and low levels of *Wnt3*, were expressed (Table 1, and Figure 1). Two of these Wnts, Wnt5a and Wnt7b, were expressed in all subregions of the hippocampus at both P7 and P14 (Figure 1C, D, G, H). Wnt7b had a particularly interesting expression pattern at P14. While still expressing in all subregions, cells in the CA2 and dentate gyrus (DG) regions had higher expression of Wnt7b. Wnt3 was only expressed in a small number of cells in the DG hilus (data not shown). The expression of Wnt7a was dynamically regulated between P7 and P14. At P7, Wnt7a was only expressed in the DG and by P14 expression of Wnt7a expanded to include both the DG and CA1 subregions (Figure 1E, F). In addition, several Wnts were also expressed in the cortex and thalamus (Additional files 1 and 2; Table 1).

**Table 1:** Wnt expression in the developing forebrain

**Gene**	**Hippocampus**	**Piriform cortex**	**Entorhinal cortex**	**Olfactory bulb**	**Cortex**	**Thalamus**
*Wnt1*	-	+	-	-	+	-
*Wnt2*	-	-	-	-	-	-
*Wnt2b*	-	-	-	-	-	+
*Wnt3*	+	-	-	-	-	+
*Wnt3a*	-	-	-	-	-	-
*Wnt4*	-	-	-		+	+
*Wnt5a*	+	+	+	+	+	+
*Wnt5b*	-	-	-	-	-	-
*Wnt6*	-	-	-	-	-	-
*Wnt7a*	+	-	-	+	-	-
*Wnt7b*	+	+	+	+	+	+
*Wnt8a*	-	-	-	-	-	-
*Wnt8b*	-	-	-	-	-	-
*Wnt9a*	-	-	-	-	-	+
*Wnt9b*	-	-	-	-	+	-
*Wnt10a*	-	-	-	-	-	-
*Wnt10b*	-	-	-	-	-	-
*Wnt11*	-	-	-	-	-	-
*Wnt16*	-	-	-	-	-	+

**Figure 2:**
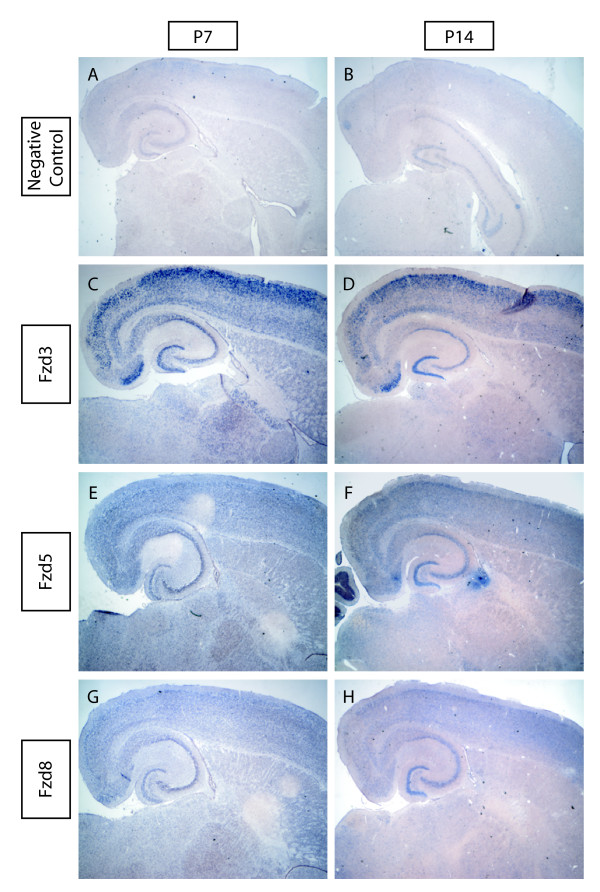
**Fzd expression in the developing hippocampus**. (A, B) Negative control *in *situ hybridizations at P7 (A) and P14 (B). (C-H) Three of six Fzds tested are expressed in the hippocampus during development of synapses. All three Fzds, Fzd3 (C, D), Fzd5 (E, F), and Fzd8 (G, H) are expressed in all three subregions at both P7 and P14. Expression was determined using 20× magnification to differentiate signal from background labeling.

To determine if hippocampal neurons express Wnt receptors, we examined the expression of the most common Wnt receptor, Fzd, by *in situ *hybridization. Using DIG-labeled probes to six of the Fzd family members, Fzd1, Fzd2, Fzd3, Fzd5, Fzd8, and Fzd9, we found three Fzds were expressed in the hippocampus during synaptogenesis (Figure 2). The three Fzds identified, Fzd3, Fzd5, and Fzd8, had consistent spatial expression patterns between P7 and P14 and were expressed in all regions (Figure 2). These results indicate that Wnts and their receptors are expressed during synapse formation and suggest that Wnt signaling might regulate the development of synapses in the hippocampus.

### Subcellular expression of Fzd3

**Figure 3:**
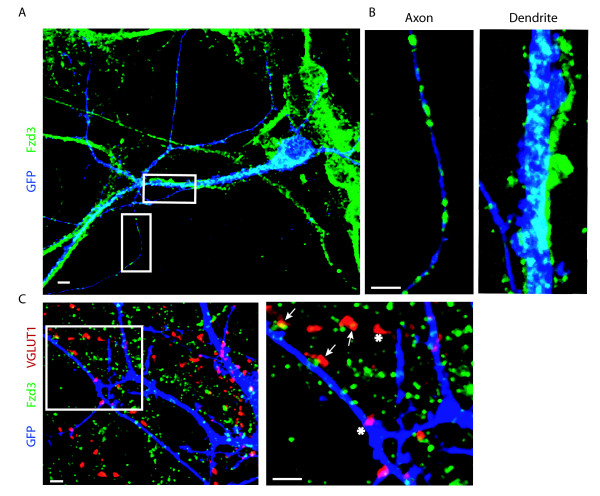
**Subcellular Fzd3 expression**. (A) An image of a green fluorescent protein (GFP) transfected neuron in a low density area stained for GFP (dark blue) and Fzd3 (green). Areas of overlap appear as light blue. (B) Sections on this neuron show that Fzd3 is expressed in both axons (B; left) and dendrites (B; right). **(C) **Staining of a GFP transfected neuron with VGLUT1 (red), Fzd3 (green), and GFP (dark blue). Some excitatory presynaptic puncta colocalize with Fzd3 (arrows) and some do not (asterisks). Scale bar = 2 μm.

We used an antibody to Fzd3 to examine the subcellular localization of Fzd3 in hippocampal cultures. Green fluorescent protein (GFP) transfected neurons were immunostained for GFP and Fzd3 at 14 days *in vitro *(14 DIV). We found that Fzd3 was heavily expressed in cell bodies, dendrites and axons (Figure 3A, B). To explore whether Fzd3 puncta were associated with presynaptic puncta, we also stained hippocampal cultures for Vesicular glutamate transporter 1 (VGLUT1) along with GFP and Fzd3. We found that Fzd3 colocalized with some VGLUT1 puncta (Figure 3C, arrows), but other VGLUT1 puncta did not colocalize with Fzd3 (Figure 3C, asterisks). These results suggest that Wnts likely act locally at presynaptic terminals and indirectly at sites along axons, dendrites, or at the soma.

### Development of hippocampal synapses in vitro

**Figure 4:**
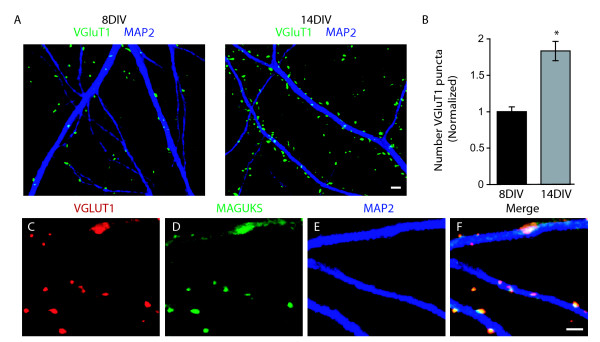
**Development of synapses in postnatal day 0 (P0) hippocampal cultures**. (A) Representative images of immunofluorescence staining of Microtubule associated protein 2 (MAP2; dark blue) and Vesicular glutamate transporter 1 (VGLUT1; green) at 8 days *in vitro *(8 DIV) (left) and 14 DIV (right). Scale bar = 2 μm. (B) Quantification of presynaptic inputs per area of dendrite at 8 DIV and 14 DIV (± standard error of the mean). To ensure equivalent staining, cells were fixed at respective days and then stained with a single reaction. The number of inputs at 14 DIV is normalized to the number of inputs at 8 DIV. n = 47 fields of view. *p < 0.001, Students *t*-test. (C-F) Colocalization of VGLUT1 puncta (C, F, red) with MAP2 (E, F, blue) and Membrane associated guanylate kinase (MAGUKs) (D, F, green). Scale bar = 2 μm.

To explore the effects of Wnts on synapse formation, we first established the time window of synaptogenesis in P0 hippocampal cultures. We immunostained cultures at 8 DIV and 14 DIV with an excitatory presynaptic marker, VGLUT1, and a dendritic marker, Microtubule associated protein 2 (MAP2; Figure 4A). We found that during the second week *in vitro*, the number of presynaptic inputs increased dramatically (Figure 4B). We also found that 85 ± 2% of VGLUT1 puncta colocalized with postsynaptic Membrane associated guanylate kinases (MAGUKs; Figure 3C–F), determined by staining with an anti-pan-MAGUK antibody. This high rate of colocalization with postsynaptic markers indicates that the number of VGLUT1 puncta associated with dendrites is representative of the number of synapses and that the increase in the number of VGLUT1 puncta between 8 DIV and 14 DIV reflects an increase in the number of synapses during this period. Consistent with this interpretation, there is a marked increase in synaptic currents in hippocampal cultures between 8 DIV and 14 DIV (EKD and AG, unpublished observation).

### Activation of the canonical Wnt pathway promotes synapse formation

The Wnts expressed in the hippocampus at the peak of synapse formation have been implicated in the activation of both canonical and noncanonical pathways. Wnt7a and Wnt7b specifically have been identified as canonical Wnt pathway activators. To verify their role as canonical pathway inducers, we examined the consequences of exposing P0 hippocampal cultures to either recombinant Wnt protein or Wnt conditioned media for 36 hours. The cellular response to different Wnts was determined using an anti-'active' β-catenin antibody that identifies non-phosphorylated β-catenin by binding to the GSK3β phosphorylation site on β-catenin [14]. In our cultures, application of LiCl, an inhibitor of GSK3β, produced a strong increase in the intensity of 'active' β-catenin in both the nucleus and soma, verifying the effectiveness of the antibody (Additional file 3).

**Figure 5:**
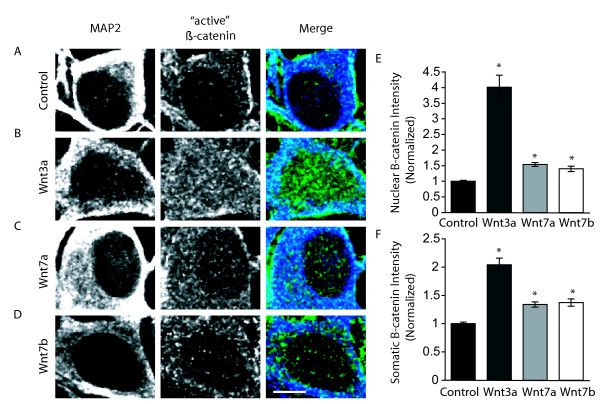
**Wnt3a, Wnt7a, and Wnt7b activate the canonical pathway in hippocampal cultures**. (A-D) Representative images of 'active' β-catenin stabilization by endogenous Wnts (A), addition of recombinant Wnt3a (B; 150 ng/mL), and addition of recombinant Wnt7a (C; 200 ng/mL), or Wnt7b conditioned media (D). Scale bar = 6 μm. (E, F) Quantification of 'active' β-catenin immunofluorescence intensity by Wnts in the nucleus (E) and soma (F). Each condition was normalized to the control condition. (n = 428 cells; *p < 0.001, *t*-test for each control and experimental group pairing, error bars represent SEM). MAP2, Microtubule associated protein 2.

We first examined the intensity of 'active' β-catenin at 10 DIV after the application of Wnt3a. Wnt3a is strongly associated with activation of the canonical pathway [15,16] and consequently served as a positive control. As expected, application of Wnt3a resulted in greater than a two-fold increase in 'active' β-catenin intensity in both the soma and the nucleus (Figure 5B, E, F). This increase in β-catenin indicates that cultured hippocampal neurons have the ability to activate β-catenin and relay a canonical Wnt signal. Application of recombinant Wnt7a or Wnt7b conditioned media also led to an increase in intensity of 'active' β-catenin immunofluorescence (Figure 5C–F), indicating that these Wnts can activate the canonical signaling pathway in hippocampal neurons. Staining results for Wnt7a purified protein application were confirmed using western blotting (Additional file 4).

**Figure 6:**
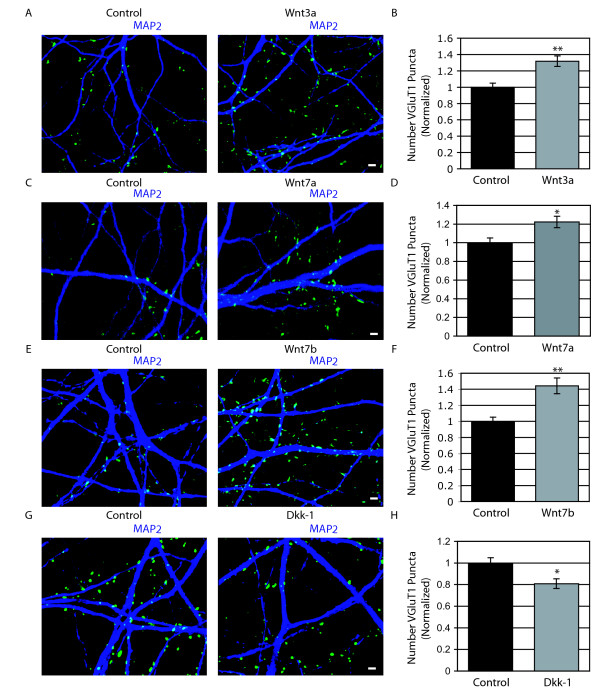
**Wnt proteins that activate β-catenin signaling increase presynaptic inputs**. (A, C, E) Representative images of control (A, C, E, left), Wnt3a (A, right), Wnt7a (C, right), or Wnt7b (E, right) treated cultures immunostained with Vesicular glutamate transporter 1 (VGLUT1; green) and Microtubule associated protein 2 (MAP2; dark blue). Cultures were treated for 36 hours and stained at 10 days *in vitro *(10 DIV). The same concentrations of proteins were used as in Figure 4. Scale bar = 2 μm. (B, D, F) Quantification of the number of VGLUT1 puncta per area of dendrite for Wnt3a (B; n = 79 fields of view; **p < 0.001, Students *t*-test), Wnt7a (D, n = 59 fields of view; **p = 0.05, Students *t*-test), or Wnt7b (F; n = 82 fields of view, **p < 0.001, Student's *t*-test) treated cultures. All conditions were normalized to control. Error bars represent SEM. (G) Representative images of control (left) and Dkk-1 (right; 1 μg/mL) treated cells stained with VGLUT1 (green) and MAP2 (dark blue) at 14 DIV after 48 hours of treatment. Scale bar = 2 μm. (H) Quantification of the number of VGLUT1 puncta per area of dendrite (± standard error of the mean). Dkk-1 treatment was normalized to control treatment. (n = 80 fields of view, *p < 0.01, Students *t*-test).

To determine if Wnt proteins that activate canonical signaling influence synapse formation, we examined the effects of exposing hippocampal cultures to recombinant Wnt proteins or Wnt conditioned media. We treated cultures at 8 DIV and examined the number of VGLUT1 puncta per area of dendrite after 36 hours. Application of Wnt3a, Wnt7a, or Wnt7b increased the number of excitatory presynaptic puncta (Figure 6A–F), suggesting that canonical Wnt signaling positively influences synapse formation.

To further explore the role of Wnt signaling in synapse formation, we examined the effects of manipulating different parts of the canonical signaling pathway. We first inhibited canonical Wnt signaling at the level of the receptor by incubating cultures with Dickkopf-1 (Dkk-1) [17]. Dkk-1 promotes internalization of the LRP5/6 coreceptor, which is required to relay a canonical signal, but not a noncanonical signal. We found that Dkk-1 destabilized β-catenin levels (Additional file 5). Addition of recombinant Dkk-1 from 12–14 DIV resulted in decreased excitatory presynaptic puncta number (Figure 6G–H), indicating that activation of the endogenous canonical pathway contributes to synapse formation.

**Figure 7:**
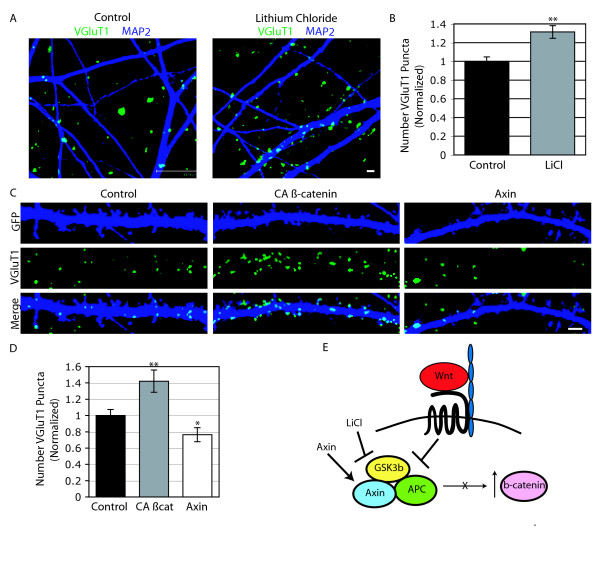
**The canonical Wnt signaling pathway increases presynaptic inputs**. (A) Representative images of Microtubule associated protein 2 (MAP2; dark blue) and Vesicular glutamate transporter 1 (VGLUT1; green) staining at 14 days *in vitro *(14 DIV) after 48 hours of treatment with NaCl (Control; left; 4 mM) or LiCl (right; 4 mM). Scale bar = 2 μm. (B) Quantification of the number of VGLUT1 puncta per area of dendrite (± standard error of the mean). LiCl treatment was normalized to the control condition (n = 58 field of views; **p < 0.001, Students *t*-test) (C) Representative images of dendrites immunostained with green fluorescent protein (GFP; dark blue) and VGLUT1 (green) at 14 DIV after transfection of GFP (left), constitutively active (CA) β-catenin (middle) or axin (right) at 7 DIV. Scale bar = 2 μm. (D) Bar histogram showing the quantification of the number of VGLUT1 puncta per area of dendrite (± standard error of the mean). All conditions were normalized to the control condition (n = 134 cells; *p < 0.05, **p = 0.001, ANOVA, then Tukey test). (E) Diagrammatic representation of the effects of Wnt, LiCl, and Axin on the regulation of β-catenin

Next, we examined the consequences of manipulating downstream canonical signaling components (Figure 7E). To mimic a canonical Wnt signal, we applied lithium chloride (LiCl) at 12 DIV for 48 hours, which leads to stabilization of β-catenin by inhibiting GSK3β [18]. Consistent with a role of canonical signaling in promoting synapse formation, we observed an increase in the number of presynaptic inputs per area of dendrite in response to LiCl treatment (Figure 7A, B). Additionally, neurons transfected with a constitutively active form of β-catenin at 7 DIV showed an increase in the number of presynaptic inputs per area of dendrite at 14 DIV (Figure 7C, middle, and 7D). Conversely, neurons transfected with full length axin, which inhibits β-catenin, decreased the number of presynaptic puncta (Figure 7C, right, and 7D). These results show that mimicking the canonical Wnt signal increases the number of presynaptic inputs and inhibiting the canonical Wnt signal decreases the number of presynaptic inputs. Taken together, these results suggest that activation of β-catenin signaling promotes synapse formation.

### Noncanonical Wnt signaling inhibits presynaptic inputs

**Figure 8:**
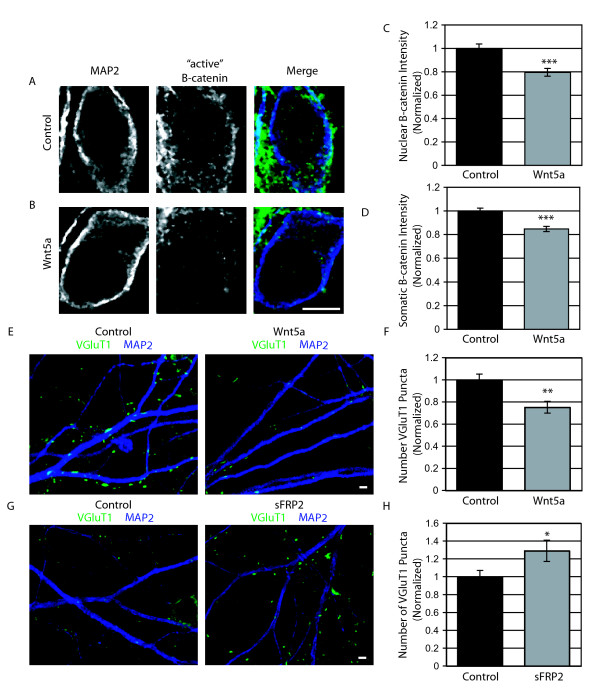
**Wnt5a acts through a non-canonical pathway and decreases the number of presynaptic inputs**. (A, B) Representative images of 'active' β-catenin immunostaining in control (A) and Wnt5a (A; 200 ng/mL) treated cells. Scale bar = 6 μm. (C, D) Quantification of 'active' β-catenin immunofluorescence intensity (± standard error of the mean) in the nucleus (C) and soma (D). Wnt5a treated cultures were normalized to the control condition (n = 220 cells, ***p < 0.001, Students *t*-test). (E) Representative images of control (left) and Wnt5a (right; 200 ng/mL) treated cells immunostained with Vesicular glutamate transporter 1 (VGLUT1; green) and Microtubule associated protein 2 (MAP2; dark blue) at 10 days *in vitro *(10 DIV). Scale bar = 2 μm. (F) Quantification of VGLUT1 puncta per area of dendrite (± standard error of the mean; n = 60 fields of view; **p = 0.001, Students *t*-test). (G) Cultures in which endogenous Wnts were inhibited with secreted Frizzled related protein 2 (sFRP2). Control (left) and sFRP2 (right; 200 ng/mL) treated cells were stained at 14 DIV with VGLUT1 (green) and MAP2 (dark blue). Scale bar = 2 μm. (H) Quantification of sFRP2 effects on the number of VGLUT1 puncta per area of dendrite (± standard error of the mean; n = 59 fields of view; *p = 0.05, Students *t*-test).

Of the Wnts expressed in the developing hippocampus, only one, Wnt5a, has been strongly implicated as a noncanonical Wnt pathway activator. However, Wnt5a has also recently been shown to signal through β-catenin [19]. Therefore, we wanted to determine whether Wnt5a behaves as a canonical or noncanonical Wnt in hippocampal cultures. We exposed cultures to recombinant Wnt5a at 8 DIV for 36 hours. We found that Wnt5a decreases 'active' β-catenin immunofluorescence intensity, indicating that Wnt5a does not activate the canonical pathway and may act as an antagonist to canonical signaling (Figure 8A–D). This decrease was confirmed by western blotting (Additional file 4).

To determine if Wnt5a affects hippocampal synapse formation, we exposed cultures to Wnt5a at 8 DIV for 36 hours and observed the effect on the number of VGLUT1 puncta. In contrast to canonical Wnts, exposure to Wnt5a led to a significant decrease in the number of presynaptic terminals (Figure 8E, F), implying that the activation of a noncanonical pathway negatively regulates synapse formation.

Finally, we wanted to examine the net effect of Wnt signaling on synapse formation given the differential effect of Wnts on VGLUT1 puncta number. To broadly inhibit Wnt signaling, we applied secreted frizzled related protein 2 (sFRP2) to hippocampal cultures. sFRP2 is an endogenous secreted protein that has a binding domain similar to Fzd [20]. We found that a two day incubation with sFRP2, at 12–14 DIV, led to an increase in excitatory presynaptic puncta number (Figure 8G, H). These results suggest that, overall, endogenous Wnts act as negative regulators of synapse formation. However, the effect of Wnts on a given cell is likely determined by the relative activation of canonical and noncanonical pathways.

## Discussion

### Expression of Wnts and Fzds in the developing forebrain

Our results indicate that several Wnts are expressed in the hippocampus during the peak of synapse formation in development. Using *in situ *hybridization, we found that of the 19 Wnt ligands, only a few are expressed in the hippocampus, including Wnt3, Wnt5a, Wnt7a, and Wnt7b. Previous studies investigating the developmental regulation of Wnt expression have shown similar results. Shimogori *et al*. [21] examined the developmental expression profile of several Wnts (1, 2b, 5a, 7a, 7b, 8b) in multiple regions of the brain. They found that Wnt5a, Wnt7a, and Wnt7b are all expressed in the hippocampus from P0 through P20, consistent with our results, although the areas showing expression are slightly different. Shimogori *et al*. [21] also reported expression of Wnt8b in the DG hilus at P20. We did not observe Wnt8b expression, but Wnt8b may start being expressed shortly after our P14 time point. Using real time PCR, Cerpa *et al*. [22] reported that in addition to Wnt5a and Wnt7a, Wnt11 and Wnt4 are expressed in hippocampal cultures at 10 DIV. We did not find any Wnt4 expression in the hippocampus, though *in situ *hybridization may not be sensitive enough to detect low signal levels from background.

We also found that three of the six Fzd receptors we tested are expressed during hippocampal development. These Fzds were Fzd3, Fzd5, and Fzd8. At present, we do not know which Fzd interacts with each Wnt, although it is likely that the Wnts present in the hippocampus bind to multiple Fzd receptors [23]. Indeed, Wnt5a has been shown to interact with nearly all of the Fzd receptors [24-27]. Additionally, we do not know which pathway each Fzd is activating. It is currently unknown how Wnts binding to different Fzd receptors create such a large variation in responses of the targeted cell, although this could be attributed to the diversity of Fzds and Wnts. Previous work has shown all three of the Fzds expressed in the developing hippocampus can activate both canonical and noncanonical pathways [24-30]. It is also possible that other receptors for Wnts are present within the developing hippocampus [31], which may mediate some of the effects of Wnt proteins in synapse formation.

We find that the Wnts expressed in the developing hippocampus have differential effects on stabilization of β-catenin. Based on our evidence of increased β-catenin levels when Wnt7a and Wnt7b is applied, and that of previous work [22,32], it is likely that Wnt7a and Wnt7b activate the canonical pathway. We also find that Wnt5a behaves in hippocampal cultures as previously described [33-36]. Because Wnt5a antagonizes the canonical pathway in our cultures, it is possible that it acts through the Wnt/Ca2+ pathway [37] or even through an alternative receptor, ROR2.

### Canonical Wnt signaling promotes synapse formation

We found that activation of the canonical Wnt pathway promotes synapse formation based on the evidence that Wnts that activate canonical Wnt signaling increase the number of presynaptic terminals per area of dendrite. The canonical pathway regulates presynaptic inputs through a series of events that begins with Wnts binding to the Fzd-LRP5/6 coreceptor, as indicated by the reduction of presynaptic inputs when this complex is inhibited by Dkk-1. The activation of the coreceptors leads to stabilization of β-catenin, indicated by the increase in presynaptic inputs when β-catenin levels are increased either by inhibition of GSK3β by LiCl or transfection of a constitutively active form of β-catenin. Additionally, when Wnt signals are blocked by transfection of axin, which is part of the complex that degrades β-catenin, we see a reduction of inputs.

There are several mechanisms by which increased β-catenin levels might promote synapse formation. First, Wnts that activate the canonical signaling pathway might act solely to stabilize β-catenin, which can in turn stabilize N-cadherin, and therefore act as part of a cell adhesion mechanism [38,39]. Second, canonical Wnts could activate the classic canonical pathway to initiate transcription of synaptic components such as EphB/EphrinB [40], which have been shown to cluster NMDA receptors [41]. Third, activation of the canonical pathway might act to localize presynaptic vesicles at the synapse through β-catenin stabilization [42]. An important goal of future experiments will be to identify the mechanisms by which β-catenin signaling promotes synapse formation.

An interesting observation is that Wnt3a activates β-catenin much more strongly than Wnt7a or Wnt7b and yet, the degree to which Wnt3a increases synapses is approximately the same as Wnt7a or Wnt7b. It therefore appears that the level of nuclear Wnt signaling is not the sole determinant of synaptogenic activity. It is likely that Wnt7a and Wnt7b activate other signaling pathways, such as the Wnt-GSK3-microtubule or Wnt-mTOR (mammalian target of rapamycin) pathways. These pathways might cooperate with the canonical signaling pathway to regulate synapse number.

### Noncanonical Wnt signaling inhibits synapse formation

In addition to effects of the canonical Wnt pathway, we also wanted to explore the effect of a noncanonical Wnt pathway on the development of synapses. We found that in hippocampal cultures, Wnt5a decreased β-catenin levels and reduced the number of presynaptic puncta. We do not yet know the pathway by which Wnt5a exerts its effect, but it is likely to be a noncanonical pathway that antagonizes the canonical pathway [4]. By antagonizing the canonical pathway, Wnt5a may inhibit any of the previously proposed mechanisms for prosynaptogenic effects of canonical pathway activation. The effects of Wnt5a on synapse number results is not a consequence of reduced axon outgrowth as preliminary data show an increase in the overlap of axons and dendrites in Wnt5a treated cultures.

Our observation that Wnt5a decreases the number of synaptic inputs differs with a study by Cerpa *et al*. [22] in which they report that Wnt5a had no effect on β-catenin or on presynaptic input number. There are several key differences between the experiments presented here and those carried out by Cerpa *et al*. [22]. The timing, duration, and type of Wnt application are vastly different between the two studies. We applied purified recombinant protein for a period of 36 hours while Cerpa *et al*. [22] applied conditioned media for several hours. Cerpa *et al*. [22] used neurons that were 14–21 DIV whereas we used more immature neurons at 8 DIV, when synapses are still forming. Studies within our lab (data not shown) show that cultures are fully mature by 21 DIV and even show elimination of synapses by this time. Therefore, the effects of the Wnts on synapse number that Cerpa *et al*. [22] were studying would be largely on the maturation of the synapses and not on the initial formation, as in our experiments.

One additional difference between our study and the study by Cerpa *et al*. [22] is the type of synaptic markers they used in quantifying synapses. They used a synaptophysin antibody that labels both inhibitory and excitatory inputs, and did not see an effect of Wnt5a, whereas we used VGLUT1, which only labels VGLUT1-positive excitatory inputs. To address this discrepancy, we stained for three different presynaptic markers, VGAT (inhibitory inputs), VGLUT1 (excitatory inputs), and Synapsin (total synaptic input). We did not find differences with application of Wnt5a on the number of Synapsin or VGAT puncta (data not shown). A lack of an effect on total Synapsin-positive puncta could be accounted for by the fact that there is an additional population of excitatory inputs that is not labeled by the VGLUT1 marker.

### Opposing roles of Wnts during synapse formation

Our results show that inhibition of endogenous Wnts by sFRP2 leads to an increase in presynaptic inputs, suggesting that Wnt5a is the dominant Wnt during synapse formation in hippocampal cultures. This could be due to higher Wnt5a expression than Wnt7a and Wnt7b combined or a higher affinity of Wnt5a for Fzd receptors, either of which would lead to more noncanonical pathway activation than β-catenin activation. While the mechanism of competition between canonical and noncanonical pathway activators is not yet known, there have been two studies that would suggest that Wnt5a is dominant over canonical Wnts. Both studies applied Wnt5a along with Wnt3a. One study found that Wnt5a lowers β-catenin levels, even when Wnt3a is added along with Wnt5a [36]. Another study shows opposing effects of Wnt5a and Wnt3a in the differentiation of stem cells. When both Wnt3a and Wnt5a are applied, Wnt5a effects are dominant [43]. Thus, we favor the hypothesis that the effects of Wnt5a are dominant over the effects of Wnt7a and Wnt7b when all are present.

Previous studies have reported divergent effects of Wnts in synapse formation. Wnts have been shown to be antisynaptogenic in *C. elegans *by a mechanism that does not involve the canonical pathway [10]. Wnt4 in *Drosophila *embryos also has an antisynaptogenic activity [11]. Although the authors do not suggest a pathway through which this is mediated, Wnt4, which is most closely related to mammalian Wnt9a and Wnt9b, has previously been described as a noncanonical Wnt [3]. Conversely, in mammals, Wnts have been shown to be pro-synaptogenic. Wnt7a, which can activate the canonical Wnt signaling, was found to alter growth cone complexity and promote synapse formation in the cerebellum [7]. Based on these observations, together with the findings presented here, we suggest that the pro- and anti-synaptogenic effects of Wnts on synapse formation are generally mediated by the level of activation of canonical Wnt signaling.

## Conclusion

The most striking observation from our experiments is that canonical Wnt pathway activation has the opposite effect on presynaptic inputs as noncanonical Wnt pathway activation. Previous work has shown Wnts to be both pro- and anti-synaptogenic, although these studies were done in several different systems with different Wnt proteins. We show here, in the same system, that Wnts exert a bidirectional influence on synapse formation.

## Materials and methods

All experiments have been carried out under the University of California, San Diego's Animal Care and Use Committee guidelines.

### Hippocampal cultures

Hippocampi were dissected from P0 Long Evans rats, dissociated, and plated onto 8-well LabTek chamber slides (Nunc, Rochester, NY, USA) coated with poly-d-lysine (Millipore, Billerica, MA, USA) and laminin (Sigma, St Louis, MO, USA) at a density of 80,000 cells/well. Cultures were grown for 8 or 12 days at 37°C with 5% carbon dioxide atmosphere. Cells were then treated with for 36 hours with lithium chloride (4 mM), sFRP2 (200 ng/mL), recombinant Dkk-1 (200 ng/mL), recombinant Wnt3a (150 ng/mL), recombinant Wnt5a (200 ng/mL), recombinant Wnt7a (200 ng/mL) or Wnt7b conditioned media. All proteins were obtained from R&D Systems (Minneapolis, MN, USA) and reconstituted in 0.1% bovine serum albumin in 1× phosphate-buffered saline (PBS). Cells treated with Wnt proteins and their control conditions were changed into 5% fetal bovine serum (FBS) media upon addition of the protein or control buffer. To control for nonspecific binding of sFRP2, heat inactivated protein was added to the control condition.

Wnt7b conditioned media was prepared by plating HEK293T cells in 60 mm dishes at a density of 2 million cells/dish in 10% FBS media. HEK293T cells were then transfected 24 hours after plating with either Wnt7b or GFP using Fugene (Roche, Mannheim, Germany). The Fugene transfectant was washed off 24 hours later and cells were changed into a 2% FBS media. HEK293T cells were incubated for 72 hours. Wnt7b or control conditioned medium was then supplemented with B27 and Glutamax (Invitrogen, Carlsbad, CA, USA), and glucose (to 120 mM) and then added directly to hippocampal cultures.

### Calcium phosphate rransfection and constructs

The constitutively active β-catenin and full length axin constructs were generously donated by Dr Tannishtha Reya. PCR amplification was used to purify the inserts with the following primer pairs: axin forward, CGC CCG CGC CCA TGC AGA GTC CCA AAA TGA ATG TCC; axin reverse, CGC CCC GGG TCA GTC CAC CTT TTC CAC CTT GCC; constitutively active β-catenin, forward, CGC GGC CGC CCA TGG CTA CTC AAG CTC ACC TGA TG; constitutively active β-catenin, reverse, CGC CCC GGG TTA CAG GTC AGT ATC AAA CCA GGC CAG. Inserts were then ligated into an IRES-GFP construct.

Constructs were transfected into hippocampal cultures at 7 DIV by calcium phosphate transfection. Briefly, low glucose DMEM (Dulbecco's modified Eagle's medium; Invitrogen, Carlsbad, CA, USA) was added to cells just before preparation of transfectant. DNA was mixed with 250 mM calcium chloride and then added to an equal volume of 2× HEBS. Twenty five microliters of transfectant solution were added to each well. Cells were incubated at 37°C for approximately 20 minutes and then washed with low glucose DMEM. DMEM was replaced with fresh media after the last wash. Cells were incubated for an additional seven days before fixation.

### Immunohistochemistry

Hippocampal cultures were briefly washed with 1× PBS and then fixed in 4% paraformaldehyde/4% sucrose solution for 20 minutes. Cells were permeated with a blocking solution of 3% bovine serum albumin and 0.1% Triton-X for 30 minutes in 1× PBS. Cells were immunolabeled with anti-'active' β-catenin (Millipore; 1:1,000), anti-VGLUT1 (Millipore; 1:5,000), anti-Fzd3 (R&D; 1:500), anti-pan MAGUK (NeuroMAb; Davis, CA, USA; 1:500), and/or anti-MAP2 (Sigma; 1:5,000) for 2 hours at room temperature or overnight at 4°C. Cultures were washed four times with blocking solution and then incubated with secondary antibodies (Jackson Immunoresearch, West Grove, PA, USA, or Molecular Probes, Eugene, OR, USA; 1:1,000) for 1 hour at room temperature. Cells were coverslipped using Fluormount-G (Southern Biotech, Birmingham, AL, USA).

### Imaging and data analysis

Z-stacks of random views of VGLUT1, MAP2, and MAGUKs or GluR2/3 immunofluorescence were captured with a Leica confocal microscope with conditions blinded to the experimenter. At least 10 images were taken per experiment and all experiments were repeated three or more times. Flattened Z stack images were first median filtered with radius of one pixel to reduce background noise and then thresholded to obtain discrete VGLUT1 puncta. The same threshold values were used throughout an entire set of images. The dendrite area was measured and the number of puncta that colocalized with the dendrite was quantified.

For β-catenin experiments, random images were taken of cell bodies stained with MAP2 and 'active' β-catenin. At least six fields of view were taken of each experiment and experiments were repeated at least two times. A region of interest was identified within the nucleus and soma for each cell and the average intensity was measured for each region of interest.

All data analysis was performed using ImageJ. Student's *t*-tests were used to determine significance between control and the experimental group. For results with more than two conditions, a single factor ANOVA was first performed and following statistical significance, a Tukey test was performed. Images prepared for figures have been enhanced in Photoshop 6.0 using a median filter to decrease background noise.

### Western blotting

Proteins from total cell extracts were separated on an 8% Bis-tris polyacrylamide gel. Blotting procedures were performed according to standard protocols and immunodection of 'active' β-catenin (1:5000) and β-tubulin (Abcam; 1:5000) was carried out.

### *In situ *hybridization

P7 and P14 mice were perfused with 4% paraformaldehyde in 0.1 M phosphate buffer. The brains were removed from the skull and post-fixed in 4% paraformaldehyde in 0.1 M phosphate buffer for 24 hours at 4°C. The tissue was then washed with 250 mM EDTA in 0.1 M phosphate buffer for 24 hours and then incubated in 25% sucrose for up to 72 hours at 4°C. Brains were embedded in TissueTek (Sakura, Torrance, CA, USA), frozen, and then stored at -80°C. Thirty micron horizontal sections were then cut using a cryostat.

*In situ *hybridization was performed on sections with a probe concentration of 600 ng/mL in hybridization solution at 58°C for 36 hours. Hybridization was followed by sequential washes with 5× (5 minutes), 2× (1 minute), 50% formamide in 0.2× (30 minutes), and 0.2× (5 minutes) SSC at 58°C. Sections were then blocked for 1 hour in 1% Blocking reagent (Roche) followed by a three hour incubation at room temperature in anti-DIG-AP (Roche; 1:3,000). Signals were detected using nitro blue tetrazolium and 5-bromo-4-chloro-3-indolyl phosphate (NBT/BCIP) for at least 2 hours and no more than 12 hours at room temperature. The hybridization signal was compared with sections using a sense probe under identical conditions. All probes were designed and made previously by the Yimin Zou lab [44].

## Abbreviations

DG: dentate gyrus; DIV: days *in vitro*; Dkk: Dickkopf; DMEM: Dulbecco's modified Eagle's medium; Dvl: Disheveled; FBS: fetal bovine serum; Fzd: Frizzled; GFP: green fluorescent protein; GSK: glycogen synthase kinase; LRP: low-density lipoprotein receptor-related protein; MAGUK: Membrane associated guanylate kinase; MAP: Microtubule associated protein; P: postnatal day; PBS: phosphate-buffered saline; sFRP: secreted frizzled related protein; VGLUT: Vesicular glutamate transporter.

## Competing interests

The authors declare that they have no competing interests.

## Authors' contributions

EKD performed all experiments. AG participated in the design of the experiments. YZ gave advice and reagents. All authors participated in the preparation of the manuscript.

## Supplementary Material

Additional file 1 **Wnt, Fzd3, and sFRP2 expression in the cortex.** A nissl stained section of the cortex is included as reference. All sections were taken from P14 animals. Click here for file

Additional file 2 **Wnt, sFRP2, and Fzd3 expression in the thalamus at P14.** A nissl stained section is included for reference. Click here for file

Additional file 3 **Lithium chloride increases 'active' β-catenin.** (A, B) Images of control (A) and LiCl (B) treated neurons stained for Microtubule associated protein 2 (MAP2) and 'active' β-catenin. Scale bar = 6 μm. (C, D) Bar histograms showing normalized intensity of 'active' β-catenin immunofluorescence increases (± standard error of the mean) in the nucleus (C) and soma (D) with lithium chloride activation. n = 131 cells, *p < 0.001, Students *t*-test. Click here for file

Additional file 4 **Wnt7a increases and Wnt5a decreases 'active' β-catenin in hippocampal cultures.** (A) Representative images of Wnt7a versus control β-catenin levels (top). B-tubulin was used as a loading control (bottom). (B) Representative images of β-catenin levels in control and Wnt5a treated conditions (top). B-tubulin was used as a loading control (bottom). (C) Bar histograms showing the relative stabilization of β-catenin in control, and Wnt7a or Wnt5a treated neurons. Error bars represent standard error of the mean. Wnt7a and Wnt5a treated conditions are normalized to control. *p < 0.01, Student's *t*-test. Click here for file

Additional file 5 **Dkk-1 decreases 'active' β-catenin.** (A, B) Representative images of control (A) and Dkk-1 (B) treated neurons stained for Microtubule associated protein 2 (MAP2; blue) and 'active' β-catenin. (C, D) Bar graphs showing normalized intensity of 'active' β-catenin immunofluorescence decreases in the nucleus (C) and soma (D). Error bars represent standard error of the mean. n = 348 cells, *p < 0.001, Student's *t*-test. Dkk-1 treatment condition was normalized to control. Click here for file
