# Supplementary figures and images for: Wnts acting through canonical and noncanonical signaling pathways exert opposite effects on hippocampal synapse formation
Source: Neural Dev. 2008 Nov 5;3:32. doi: 10.1186/1749-8104-3-32 (PMC2596118; doi:10.1186/1749-8104-3-32)

P14 Cortex

|                                                                                     |                                                                                    |                                                                                      |
|-------------------------------------------------------------------------------------|------------------------------------------------------------------------------------|--------------------------------------------------------------------------------------|
| Nissl                                                                               | Wnt1                                                                               | Wnt4                                                                                 |
| 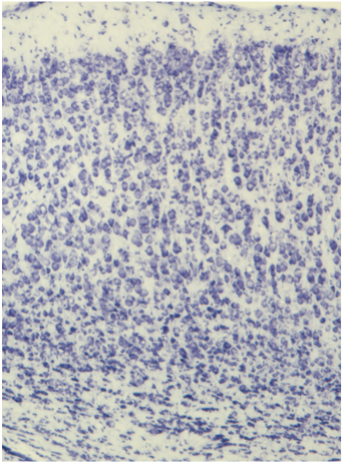   | 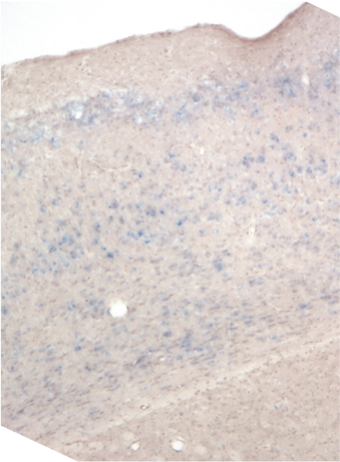  | 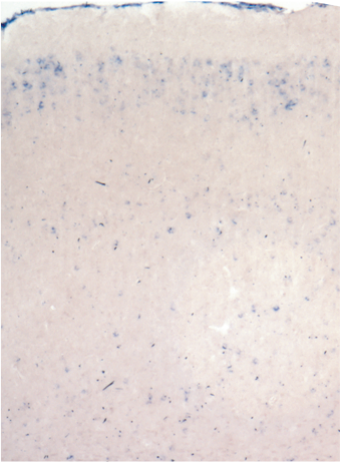  |
| Wnt5a                                                                               | Wnt7b                                                                              | Wnt9a                                                                                |
| 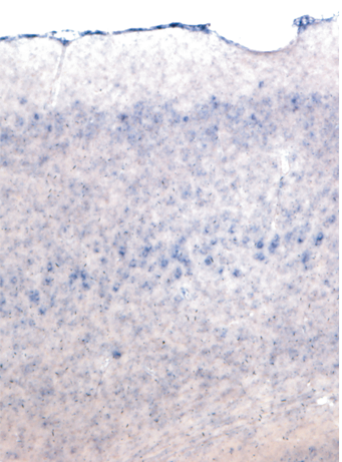  | 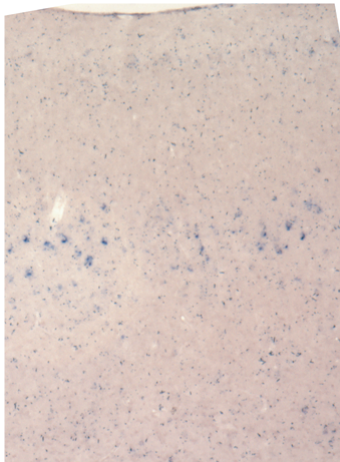 | 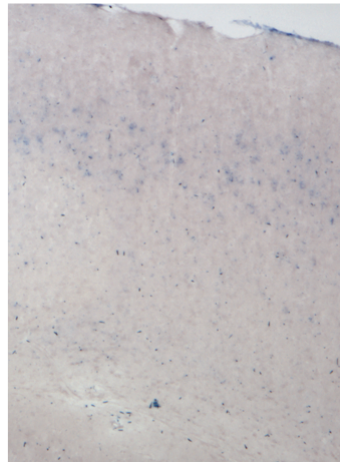 |
| sFRP2                                                                               |                                                                                    |                                                                                      |
| 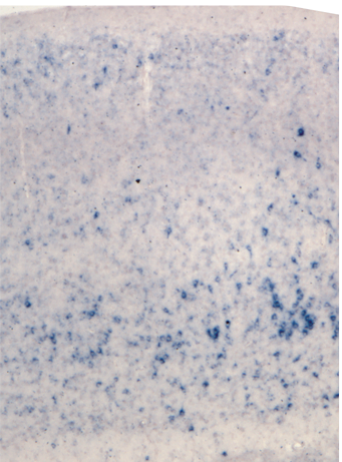 |                                                                                    |                                                                                      |

Supplement: Additional file 1 — Wnt, Fzd3, and sFRP2 expression in the cortex. A nissl stained section of the cortex is included as reference. All sections were taken from P14 animals. [file 1749-8104-3-32-S1.pdf]

# P14 Thalamus

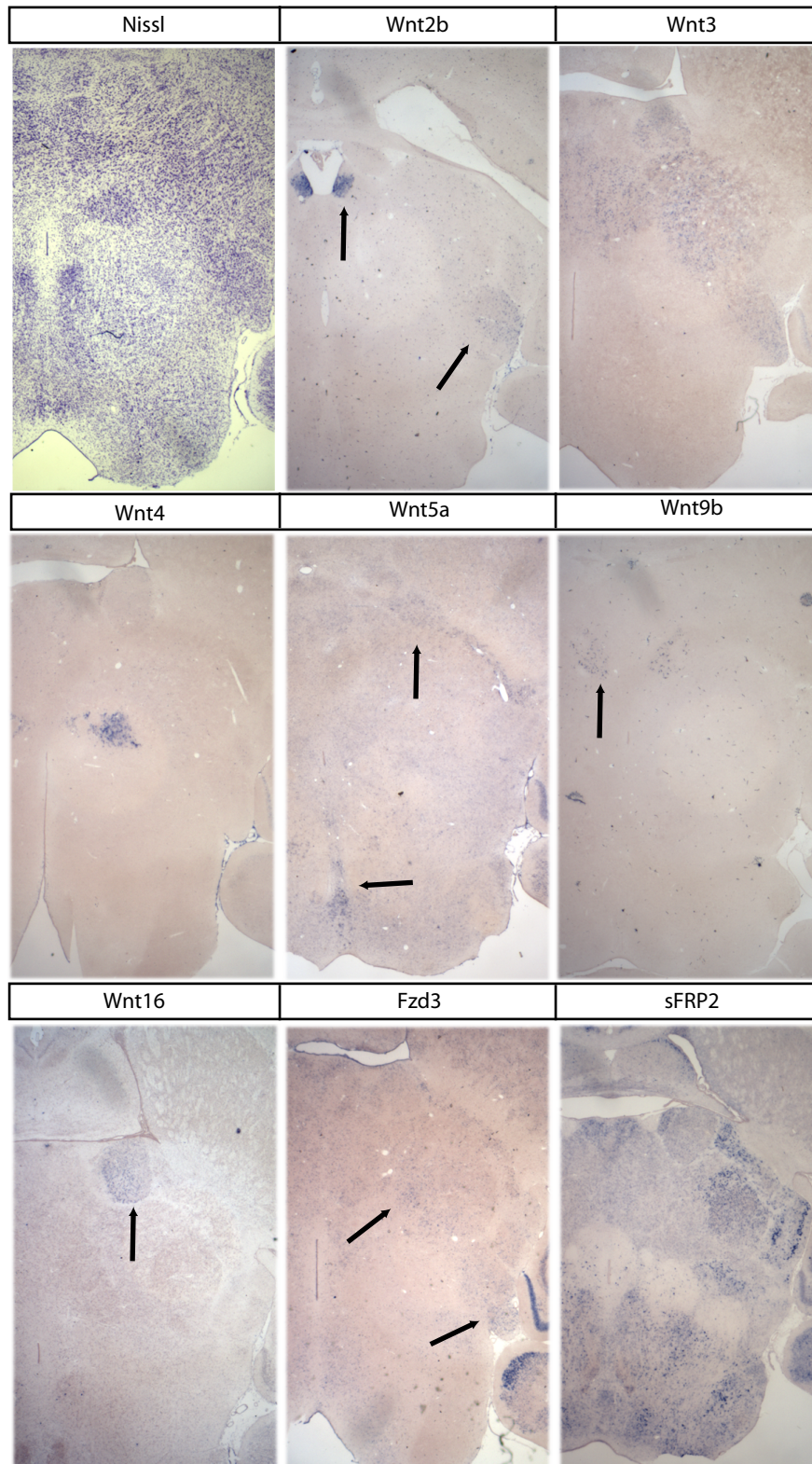

Supplement: Additional file 2 — Wnt, sFRP2, and Fzd3 expression in the thalamus at P14. A nissl stained section is included for reference. [file 1749-8104-3-32-S2.pdf]

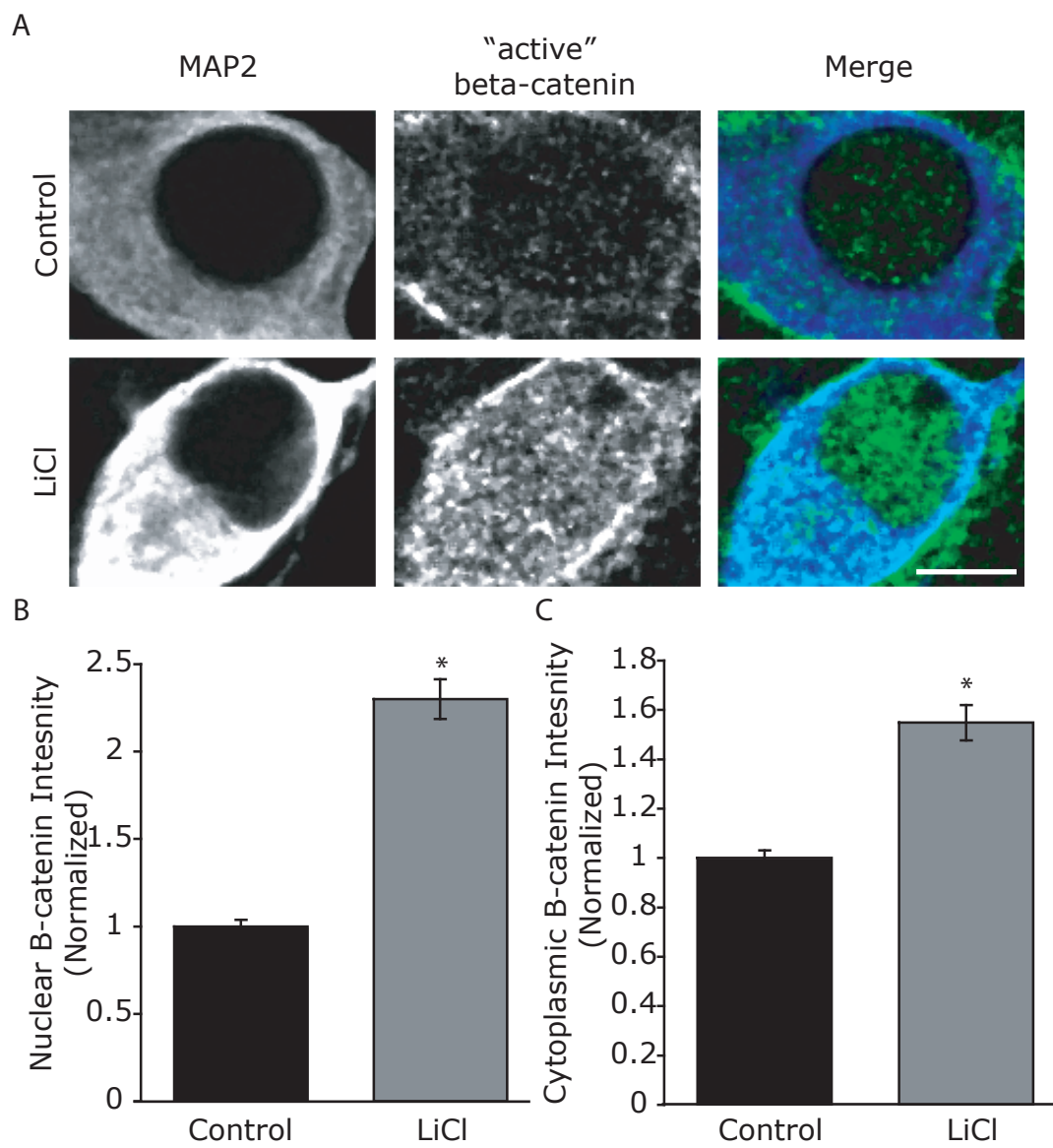

Supplement: Additional file 3 — Lithium chloride increases 'active' β-catenin. (A, B) Images of control (A) and LiCl (B) treated neurons stained for Microtubule associated protein 2 (MAP2) and 'active' β-catenin. Scale bar = 6 μm. (C, D) Bar histograms showing normalized intensity of 'active' β-catenin immunofluorescence increases (± standard error of the mean) in the nucleus (C) and soma (D) with lithium chloride activation. n = 131 cells, *p < 0.001, Students t-test. [file 1749-8104-3-32-S3.pdf]

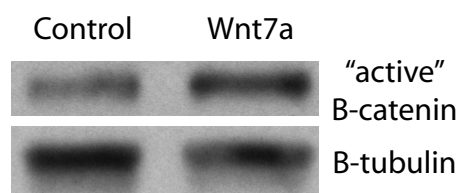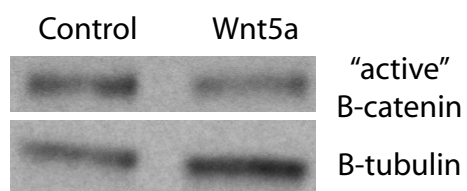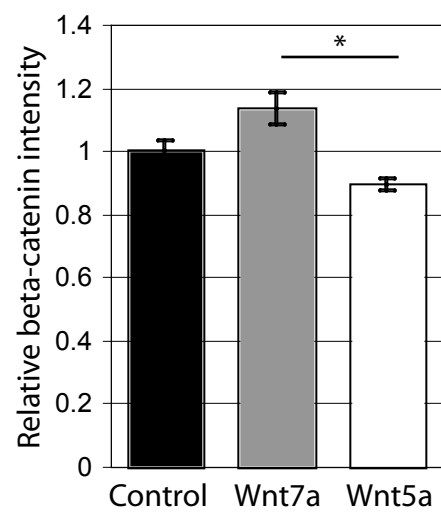

Supplement: Additional file 4 — Wnt7a increases and Wnt5a decreases 'active' β-catenin in hippocampal cultures. (A) Representative images of Wnt7a versus control β-catenin levels (top). B-tubulin was used as a loading control (bottom). (B) Representative images of β-catenin levels in control and Wnt5a treated conditions (top). B-tubulin was used as a loading control (bottom). (C) Bar histograms showing the relative stabilization of β-catenin in control, and Wnt7a or Wnt5a treated neurons. Error bars represent standard error of the mean. Wnt7a and Wnt5a treated conditions are normalized to control. *p < 0.01, Student's t-test. [file 1749-8104-3-32-S4.pdf]

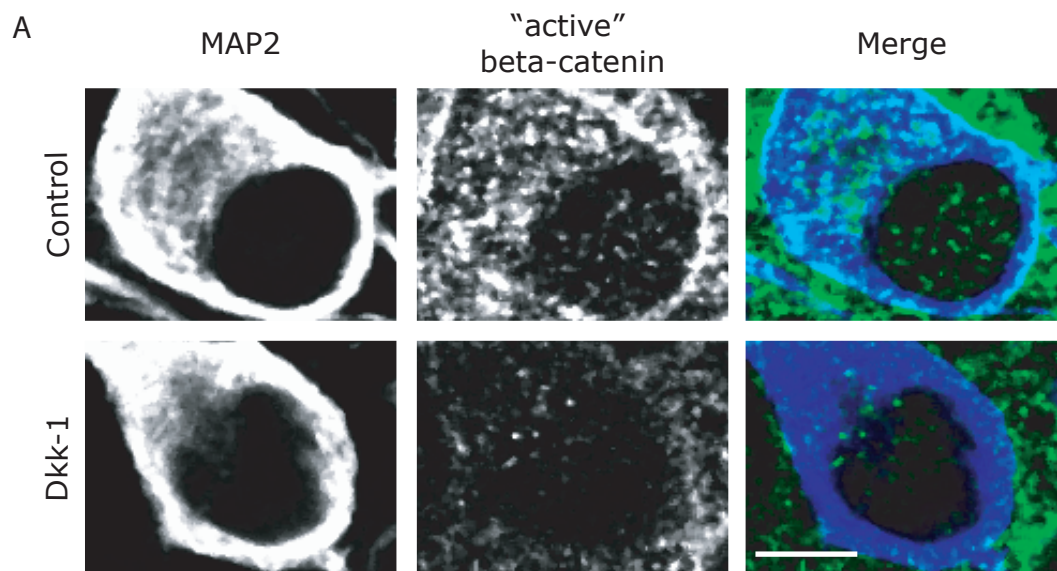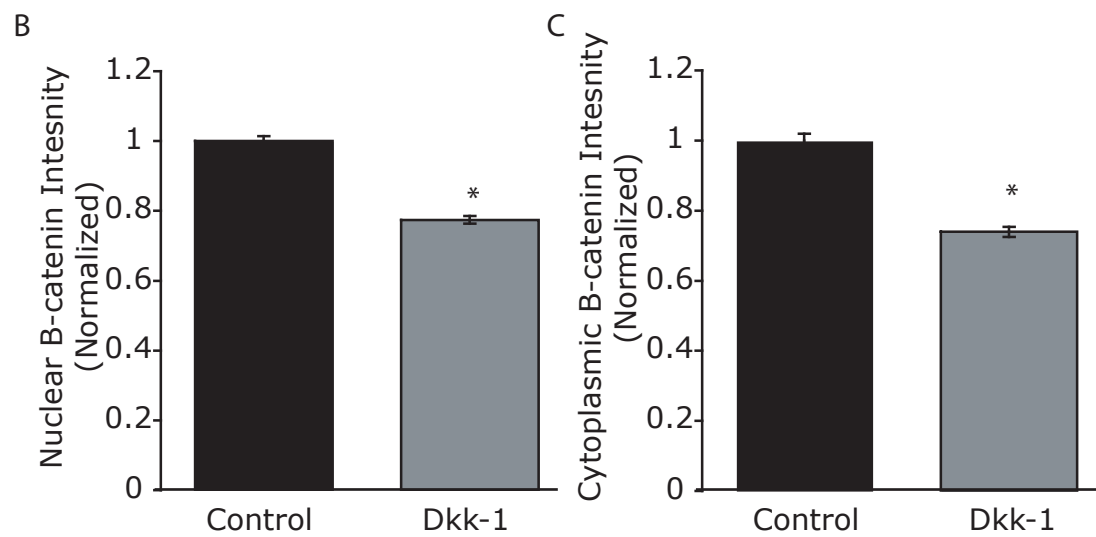

Supplement: Additional file 5 — Dkk-1 decreases 'active' β-catenin. (A, B) Representative images of control (A) and Dkk-1 (B) treated neurons stained for Microtubule associated protein 2 (MAP2; blue) and 'active' β-catenin. (C, D) Bar graphs showing normalized intensity of 'active' β-catenin immunofluorescence decreases in the nucleus (C) and soma (D). Error bars represent standard error of the mean. n = 348 cells, *p < 0.001, Student's t-test. Dkk-1 treatment condition was normalized to control. [file 1749-8104-3-32-S5.pdf]
